# Linifanib (ABT-869) Potentiates the Efficacy of Chemotherapeutic Agents through the Suppression of Receptor Tyrosine Kinase-Mediated AKT/mTOR Signaling Pathways in Gastric Cancer

**DOI:** 10.1038/srep29382

**Published:** 2016-07-08

**Authors:** Jing Chen, Jiawei Guo, Zhi Chen, Jieqiong Wang, Mingyao Liu, Xiufeng Pang

**Affiliations:** 1 Shanghai Key Laboratory of Regulatory Biology, Institute of Biomedical Sciences and School of Life Sciences, East China Normal University, Shanghai 200241, China; 2 Key Laboratory of Reproduction and Genetics in Ningxia, Ningxia Medical University, Yinchuan 750004, China; 3 Cancer Institute, Fudan University Shanghai Cancer Center; Department of Oncology, Shanghai Medical College, Fudan University, Shanghai 200032, China; 4 Institute of Biosciences and Technology, Department of Molecular and Cellular Medicine, Texas A&M University Health Science Center, Houston, Texas 77030, USA

## Abstract

Gastric cancer, highly dependent on tumor angiogenesis, causes uncontrolled lethality, in part due to chemoresistance. Here, we demonstrate that linifanib (ABT-869), a novel multi-targeted receptor tyrosine kinase inhibitor, markedly augments cytotoxicity of chemotherapies in human gastric cancer. ABT-869 and chemotherapeutic agents exhibited a strong synergy to inhibit the viability of several gastric cancer cell lines, with combination index values ranging from 0.017 to 0.589. Additionally, the combination of ABT-869 and chemotherapeutic agents led to remarkable suppression of vascular endothelial growth factor (VEGF)-induced angiogenesis *in vitro* and *in vivo*. Importantly, in a preclinical gastric cancer xenograft mouse model, drug co-treatments led to increased mouse survival as well as a synergistic reduction in tumor size and the inhibition of tumor angiogenesis. Mechanistic studies further revealed that all of the co-treatments containing ABT-869 resulted in decreased activation of the VEGF receptor, the epidermal growth factor receptor and the insulin growth factor receptor. Inhibition of these receptor tyrosine kinases consequently attenuated the activation of the downstream AKT/mTOR signaling pathway both in cultured gastric cancer cells and in gastric cancer xenografts. Collectively, our findings suggest that the addition of ABT-869 to traditional chemotherapies may be a promising strategy for the treatment of human gastric cancer.

Gastric cancer is the fourth most commonly diagnosed cancer and the second most common cause of cancer-related death worldwide. It is a heterogeneous disease with diverse molecular and histological subtypes. Despite improvements in surgical and chemotherapy approaches as well as its declining incidence, gastric cancer remains a major global public health problem. Most recently, whole-genome sequencing studies revealed that several new driver genes significantly mutated in gastric cancer, including *RHOA*, *MUC6* and *CTNNA2*^1^. Nevertheless, only a few of these somatic alterations (such as ERBB2, ERBB3 and PI3K/AKT/mTOR) are being pursed clinically. Fluoropyrimidine- or platinum-based conventional cytotoxic chemotherapy is still widely used as the first-line treatment for gastric cancer^2^,^3^. Unfortunately, resistance to these agents as well as acquired multi-drug resistance with high adverse effects invariably occurs, thus limiting the efficacy of this approach. Identification of efficacious therapeutic interventions that sensitize gastric cancer cells to conventional treatments is highly desirable, and such strategies should have a significant impact on clinical settings.

One successful treatment strategy for gastric cancer is the inhibition of angiogenesis. In addition to interrupting the supply of oxygen and nutrients in tumors, anti-angiogenesis therapy alone or in combination with other treatments transiently normalizes the structure and function of tumor vasculatures^4^. Anti-angiogenic agents can be used to induce the remodeling of tumor vasculature, leading to a temporary improvement in tumor perfusion and re-oxygenation that potentially enhance the actions of chemotherapeutic agents. We previously showed that high vascular endothelial growth factor (VEGF) levels in the tumor and serum were associated with poor prognosis in both resectable and advanced gastric cancer^5^. Clinically, the addition of bevacizumab (a humanized anti-VEGF monoclonal antibody) or sorafenib (a small molecule tyrosine kinase inhibitor) to standard chemotherapy enhanced survival of advanced gastric cancer patients^6^,^7^. These impressive results indicate that the combinations of anti-angiogenic inhibitors and chemotherapeutic agents are potentially effective to treat gastric cancer.

Functionally similar to sorafenib, ABT-869 is a novel orally available multi-target receptor tyrosine kinase (RTK) inhibitor. ABT-869 inhibits both the VEGF and platelet-derived growth factor (PDGF) receptor families, and it exhibits potent anti-angiogenic and antitumor effects^8^. ABT-869 is currently in active preclinical and clinical cancer therapeutic development. To date, tumor regression in response to ABT-869 has been evaluated in multiple xenograft models^9^,^10^,^11^,^12^. Moreover, the clinical benefits of ABT-869 have been assessed in phase I to III trials in patients with non-small cell lung cancer, acute myeloid leukemia, colorectal cancer and hepatocellular carcinoma^13^. These trials have provided evidences for the safety and efficacy of ABT-869 treatment. However, the efficacy of ABT-869 in gastric cancer has not been evaluated yet. Here we test whether ABT-869 augments the efficacy of conventional chemotherapies in preclinical *in vitro* and *in vivo* gastric cancer models.

Our results suggest that ABT-869, when given in combination with subtoxic concentrations of 5-Fu or cisplatin, shows significant anticancer effects with no signs of toxicity *in vivo*. These effects are mediated through targeting the receptor tyrosine kinases (VEGFR, EGFR and IGFR) and the AKT/mammalian target of rapamycin (mTOR) signaling cascade. This combination regimen holds promise as a potential therapeutic alternative for the treatment of patients with gastric cancer that is refractory to standard chemotherapy.

## Results

### Cytotoxic synergy of ABT-869 and chemotherapeutic agents

The cytotoxic effect of ABT-869 as a single agent was first evaluated in a wide spectrum of human gastric cancer cell lines. ABT-869 (chemical structure is shown in Supplementary Figure S1a) inhibited cell growth in a dose-dependent manner, with the half maximal inhibitory concentration (IC_50_) values ranging from 2.6 to 16.9 μmol/L (Supplementary Figure S1b). Notably, normal human gastric epithelial GES-1 cells were much resistant to ABT-869 (IC_50_ > 100 μmol/L), suggesting that the use of ABT-869 in cancer treatment may be safe and specific to tumor cells. We also examined the cytotoxicity of cisplatin in a chemo-resistant gastric cancer cell line and found that SGC-7901/DDP cells were more than 10-fold resistant to cisplatin.

**Figure 1:**
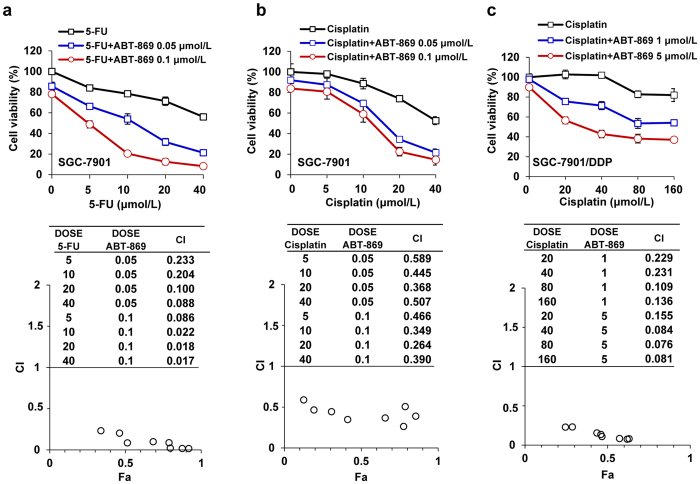
ABT-869 synergizes with chemotherapies in gastric cancer cells. Gastric cancer cells were incubated with increasing concentrations of different drugs for 48 h, followed by cell viability detection. SGC-7901 cells were treated with drug pair of ABT-869 and 5-Fu (**a**) or ABT-869 and cisplatin (**b**). Cisplatin-resistant SGC-7901/DDP cells were treated with ABT-869 and cisplatin (**c**). The drugs were added at various ratios as indicated in the Fa-CI plots. The combinatorial index values were calculated using CalcuSyn software. Fa represents the fraction affected by the dose. *Dots*, the means of five independent replicates; *bars*, standard deviation.

To evaluate the interaction between ABT-869 and 5-Fu or cisplatin, we examined the effect of drug co-treatments in gastric cancer SGC-7901cells and SGC-7901/DDP cells. The treatment of SGC-7901 cells with the combination of a minimally toxic dose of ABT-869 (0.05 and 0.1 μmol/L) and graded doses of 5-Fu (Fig. 1a) or cisplatin (Fig. 1b) resulted in decreased cell viability compared with each single agent alone. Importantly, similar results were also observed in chemo-resistant SGC-7901/DDP cells (Fig. 1c). We used the Fa-CI plots to determine the drug effectiveness and combination index (CI) values. The calculated CI values of all of the gastric cancer cell lines were less than 1 (ranging from 0.017 to 0.589), suggesting that ABT-869 and chemotherapies (either 5-Fu or cisplatin) produces a strong synergic effect in both SGC-7901 cells and SGC-7901/DDP cells.

To explore the most effective drug combinations with the highest safety margin, we used various dilutions of ABT-869, 5-Fu and cisplatin with concentrations below their IC_50s_ *in vitro*. For the initial cytotoxic screens, we chose the following drug concentrations for pair-wise treatments in SGC-7901 and MGC-803 cells: ABT-869, 0.05, 0.1 μmol/L; 5-Fu, 10 μmol/L; and cisplatin, 15 μmol/L. We treated cisplatin-resistant gastric cancer SGC-7901/DDP cells with either 1 or 5 μmol/L of ABT-869 and 40 μmol/L of cisplatin.

### ABT-869 potentiates chemotherapy-induced cell cycle arrest and apoptosis

**Figure 2:**
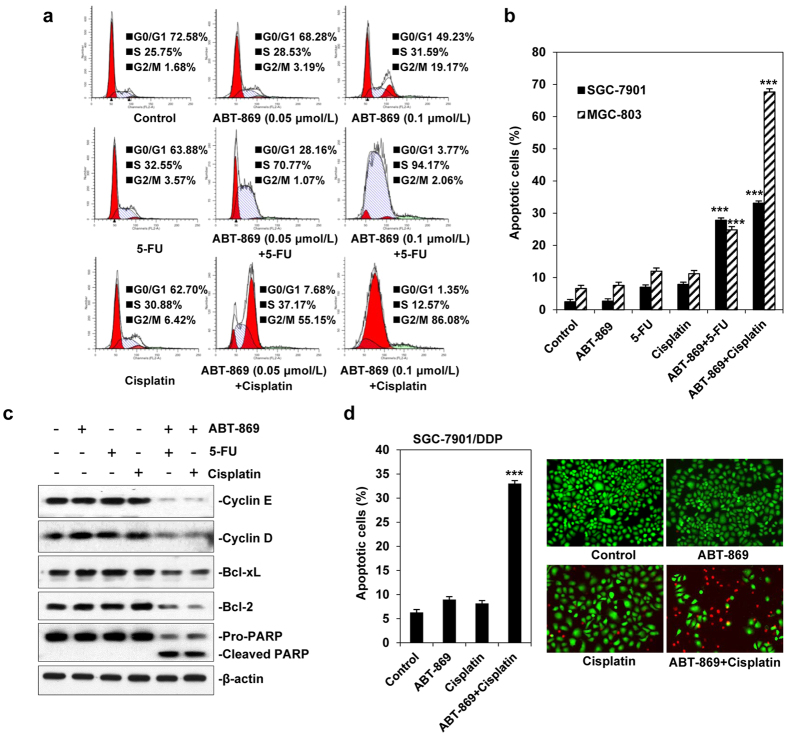
ABT-869 potentiates cell cycle arrest and apoptosis mediated by 5-Fu or cisplatin. **(a**) Treatment with ABT-869 enhanced the cell cycle arrest triggered by 5-Fu or cisplatin. SGC-7901 cells were treated with the indicated concentrations of different agents alone or in combination for 48 h, followed by cell cycle analysis. (**b**) ABT-869 strengthened the apoptotic effects of 5-Fu or cisplatin. SGC-7901 and MGC-803 cells were treated with ABT-869 (0.1 μmol/L), 5-Fu (10 μmol/L) and cisplatin (15 μmol/L) either alone or in combination for 48 h. The percentage of apoptotic cells was determined by Annexin-V and propidium iodide staining. (**c**) ABT-869-containing treatments resulted in increased cell cycle arrest and apoptosis, as indicated by specific molecular markers. The cell lysates of drug-treated SGC-7901 cells were probed for cyclin E, cyclin D, Bcl-xL, Bcl-2 and cleaved PARP by Western blotting assays. (**d**) ABT-869 promoted cisplatin-mediated cell death in a cisplatin-resistant cell line. SGC-7901/DDP cells were treated with ABT-869 (0.1 μmol/L) and cisplatin (40 μmol/L) alone or in combination. Cell apoptosis analysis and live/dead staining were performed. The live cells were stained green and dead cells were stained red. Representative images are shown. *Columns*, means; *bars*, standard deviation. ***P < 0.001 *vs.* 5-FU or cisplatin alone group.

To further explore the synergistic toxicity between ABT-869 and chemotherapies, we examined cell cycle arrest and apoptosis using flow cytometry. A number of studies have demonstrated that ABT-869 might induce either G0/G1 or S phase arrest^14^, whereas 5-Fu and cisplatin were found to induce S and G2/M phase arrest, respectively^15^,^16^. Compared with single agent alone, treatment with 5-Fu at the designated concentrations in the presence of a minimally toxic concentration of ABT-869 (e.g., 0.05 or 0.1 μmol/L) induced a significant increase in the number of cells that were in the S phase. Similarly, the combination of ABT-869 and cisplatin caused an almost complete arrest of cells in the G2/M phase (Fig. 2a). The cell cycle analysis in MGC-803 cells (Supplementary Figure S2) and BGC-823 cells (Supplementary Figure S3) was consistent with that in SGC-7901 cells. The pro-apoptotic effects of dual treatments were next examined. As shown in Fig. 2b, ABT-869 in combination with either 5-Fu or cisplatin induced a significant increase in apoptosis compared with 5-Fu or cisplatin alone group in both SGC-7901 and MGC-803 cells (*P* < 0.001). Similarity was also shown in AGS and BGC-823 cells (Supplementary Figure S4). To further investigate the inhibition of cell growth, we performed Western blotting assays to determine the mechanism of cell cycle arrest and apoptosis. Consistent with our observations, there was an increase in the level of cleaved PARP and a dose-dependent reduction in Bcl-2, Bcl-xL and CyclinD1 in co-treated SGC-7901 cells (Fig. 2c). Where noted, a significant synergism of pro-apoptotic action by drug pairs was also observed in SGC-7901/DDP cells (*P* < 0.001; Fig. 2d). These results suggest that ABT-869 sensitizes chemotherapeutic agents and may be useful for the treatment of human gastric cancer.

### Molecular basis of the effects of ABT-869 in combination with 5-Fu or cisplatin

**Figure 3:**
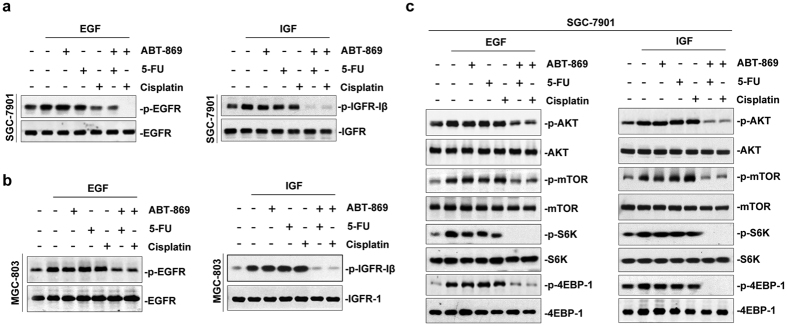
The combined regimens decrease the RTK-mediated AKT/mTOR pathways. **(a**,**b**) ABT-869 treatment blocked the growth factor-induced activation of EGFR and IGFR in gastric cancer cells. Serum starved (2% FBS, overnight) SGC-7901 cells (**a**) or MGC-803 cells (**b**) were pre-treated with ABT-869, 5-Fu and cisplatin alone or in combination for 24 h. The cells were then treated with EGF (50 ng/mL) or IGFII (50 ng/mL) for an additional 10–15 min. EGFR and IGFR phosphorylation was examined by Western blotting assays. (**c**) Treatment with ABT-869 blocked growth factor-driven activation of the AKT/mTOR signaling pathway in SGC-7901 cells. The cells were treated as described above and lysates were analyzed by Western blotting assays.

To delineate the molecular mechanism by which the combination of ABT-869 and chemotherapies inhibited tumor growth, we analyzed the key signaling molecules and pathways in drug-treated gastric cancer cells. Given that ABT-869 could inhibit multiple receptor tyrosine kinases, we first examined whether the dual treatments of ABT-869 and chemotherapies (5-Fu and cisplatin) could effectively inhibit the specific tyrosine kinase receptors that were overexpressed in gastric cancer. As shown in Fig. 3a (*left)*, EGF-induced EGFR phosphorylation at Tyr1068 site was largely inhibited by the combined treatments, whereas no significant change of EGFR activation was observed in SGC-7901 cells treated with single agent alone. Notably, similar results were found for IGF1R activation (Fig. 3a, *right*). Additionally, we found that drug co-treatment also led to a significant decrease in EGFR and IGF1R levels in MGC-803 cells (Fig. 3b). These data suggested that ABT-869 played a critical role in the chemo-sensitization of gastric cancer. As a consequence of receptor inactivation, the combined treatment suppressed the phosphorylation of AKT and mTOR as well as the downstream effectors of the AKT-mTORC1 signaling such as 4EBP and P70S6K (Fig. 3c). These results suggest that the inactivation of EGFR and IGF1R is sufficient to block the major downstream survival signaling pathways. ABT-869 and 5-Fu or cisplatin lead to synergistic effects at molecular levels.

### Synergistic effects of ABT-869 and chemotherapies on angiogenesis inhibition *in vitro* and *in vivo*

**Figure 4:**
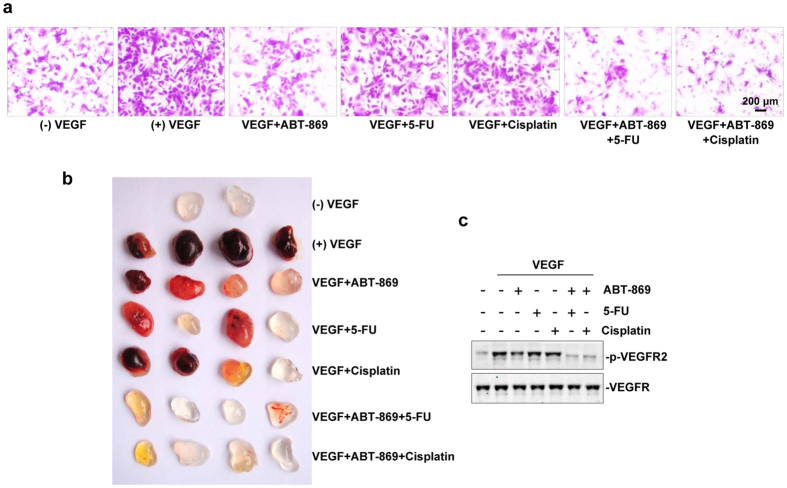
The combinations of ABT-869 and chemotherapies block VEGF-induced angiogenesis *in vitro* and *in vivo*. **(a**) ABT-869 inhibited the VEGF-induced migration of endothelial cells. Serum starved (0.5% FBS, overnight) HUVEC cells were exposed to ABT-869 (0.1 μmol/L), 5-Fu (10 μmol/L) and cisplatin (15 μmol/L) alone or in combination for 30 min. Cells were then seeded into chambers surrounded by plain endothelial cell growth medium or endothelial cell growth medium containing VEGF (50 ng/ml). After 6 h, the cells were fixed and stained with 1% crystal violet. Representative photographs were chosen from four random fields (magnification, 100x). **(b)** ABT-869 inhibited angiogenesis in a Matrigel plug assay. Six-week-old nude mice were injected with 0.4 mL of Matrigel containing ABT-869 (0.1 μmol/L) and/or 5-Fu (10 μmol/L) and/or cisplatin (15 μmol/L) in the presence of 50 ng of VEGF or 20 units of heparin. After 7 d, the skin of each mouse was pulled back to expose the intact Matrigel plugs. Representative Matrigel plugs were photographed. (**c**) ABT-869 treatment suppressed the VEGF-induced activation of VEGFR2. Serum starved (0.5% FBS, 6 h) HUVEC cells were exposed to ABT-869 (0.1 μmol/L), 5-Fu (10 μmol/L) and cisplatin (15 μmol/L) alone or in combination for 1 h. Cells were then harvested and analyzed by Western blotting assays.

Given that ABT-869 is an angiogenesis inhibitor, we examined whether the migratory potential of HUVECs could be affected by the treatment of ABT-869 combined with traditional chemotherapies. In the Boyden chamber assay, HUVEC migration was not markedly affected by 5-Fu or cisplatin, whereas ABT-869 alone caused a partial inhibition (40–50%) of endothelial cell migration (Fig. 4a). Interestingly, the combined treatment of ABT-869 and chemotherapies produced a dramatic suppression (80–90%) of migratory potential of HUVECs. We further explored the anti-angiogenic activity of the combined regimens using an *in vivo* angiogenesis model, the Matrigel plug assay. As shown in Fig. 4b, Matrigel plugs containing VEGF alone appeared dark red, indicating that functional vasculatures had formed inside the Matrigel through angiogenesis triggered by VEGF. Consistent with our *in vitro* findings, drug co-treatments dramatically inhibited VEGF-induced vascular formation, since the plugs treated with ABT-869 plus 5-Fu or ABT-869 plus cisplatin were much paler in color. Furthermore, we found that the synergistic effects of these drug combinations on angiogenesis inhibition were likely due to simultaneous inhibition of the VEGF/VEGFR2-dependent pathway in HUVECs (Fig. 4c).

### Addition of ABT-869 to chemotherapies leads to enhanced inhibition of tumor growth and increased mouse survival *in vivo*

**Figure 5:**
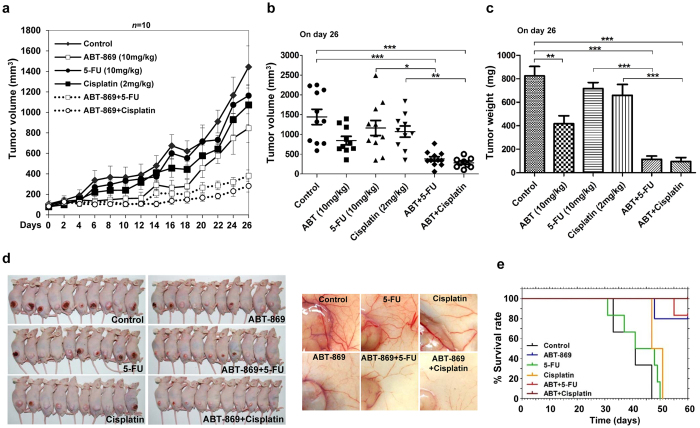
ABT-869 enhances the efficacy of chemotherapeutic agents in a gastric cancer xenograft mouse model. SGC-7901 cancer cell subcutaneous xenografts were initiated in BALB/c nude mice (*n* = 10–11) and the mice were treated with ABT-869 (oral gavage, 10 mg/kg/day) and/or 5-Fu (i.p., 10 mg/kg/day) and/or cisplatin (i.p., 2 mg/kg/day) at five-day intervals for eight cycles. An equivalent amount of pure refined corn oil or dimethyl sulfoxide served as a vehicle control. (**a**) Tumor growth curves. (**b**) Tumor volume on day 26. (**c)** Tumor weight on day 26. (**d)** Representative photographs of tumor-bearing mice (*Left*) and representative photographs of tumor angiogenesis (*right*). (**e)** Combination therapies with ABT-869 prolonged mouse survival. Kaplan-Meier survival analysis was performed in mice whose tumor volumes reached over 1,800 mm^3^. Statistical comparisons were performed by One-way ANOVA analysis. *Columns* and *dots*, means; *bars*, standard deviation. *P < 0.05; **P < 0.01; ***P < 0.001.

To expand our preclinical findings *in vivo*, we next investigated the antitumor activity of ABT-869 and chemotherapeutic agents in nude mice bearing subcutaneous human gastric cancer xenografts. As shown in Fig. 5a, the growth of SGC-7901 xenografts was not suppressed by either 5-Fu or cisplatin as a single agent at tested dosage, whereas long-lasting tumor regression occurred in the dual-treatment groups (*P* < 0.001; Fig. 5a). Specifically, 90% of the mice given combined treatments experienced complete tumor shrinkage as indicated by tumor volume (Fig. 5b) and tumor weight (Fig. 5c) at the end of the experiment. Moreover, when the skin of each mouse was pulled back to expose intact tumors, we found that the tumor growth suppression mediated by the drug co-treatments correlated with the level of angiogenesis inhibition (Fig. 5d). As a surrogate marker for survival, each mouse was sacrificed when its tumor reached approximately 1,800 mm^3^ in any one dimension. Survival curves using Kaplan-Meier analysis showed that the combined therapies significantly improved overall survival compared with single-agent or vehicle treatment (Fig. 5e). Eighty-three percentages of the mice treated with ABT-869 plus 5-Fu and all of the mice treated with ABT-869 plus cisplatin survived for more than 60 days. Importantly, our study showed that combining ABT-869 with chemotherapies (either 5-Fu or cisplatin) was well tolerated in mice using the dosage and treatment schedule described above, with no weight loss or other noticeable signs of systemic toxicity.

### Mechanistic analysis of xenograft tumors

**Figure 6:**
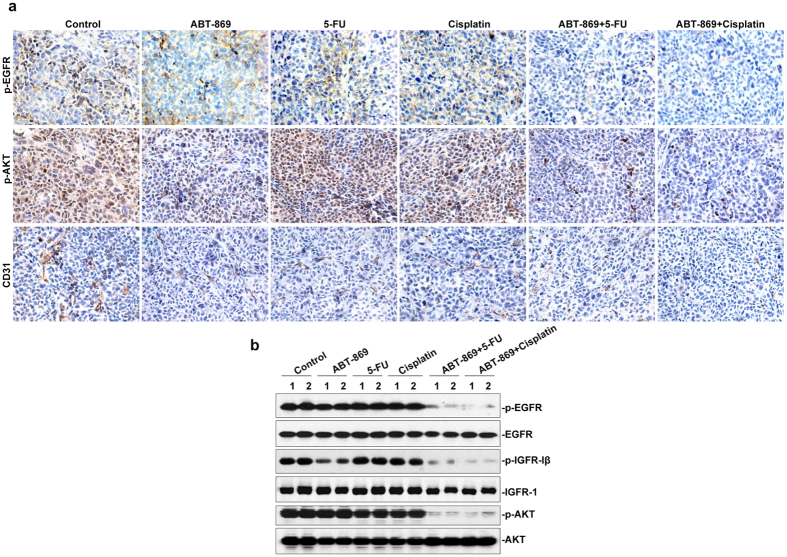
The combinations of ABT-869 and chemotherapies block the RTK/AKT pathway in gastric cancer xenografts. **(a)** Immunohistochemical staining of p-EGFR, p-AKT and CD31. Solid tumors from different treatments were collected and immunohistochemistry was performed. Three tumors were randomly selected from different groups and representative images were shown (magnification, 400x). (**b**) Western blotting analysis of proteins in the RTK/AKT pathway was performed in xenograft lysates. Two tumors were randomly selected from six different groups, and the experiments were repeated three independent times with consistent results.

To validate the molecular basis of the synergy of ABT-869 and chemotherapies, we performed immunohistochemistry and Western blotting assays on the tumor xenografts. As shown in Fig. 6a, the levels of phosphorylated EGFR and AKT were suppressed by dual treatments. Additionally, the number of CD31-positive endothelial cells in the combined treatment groups was significantly decreased compared to that from either the vehicle control group or single-agent groups (Fig. 6a), suggesting a remarkable reduction of tumor neovascularization.

Western blotting analysis of protein from randomly selected tumors showed that the activity of both EGFR- and IGFR-mediated AKT signaling pathways was inhibited by drug co-treatments. These pathways were strongly suppressed by the addition of ABT-869 (Fig. 6b). In contrast, no remarkable inhibition of this pathway was observed when either 5-Fu or cisplatin was used as a single agent. All of these mechanistic results were consistent with the *in vitro* data shown in Fig. 3.

## Discussion

5-Fu and cisplatin are currently used as first-line chemotherapy for gastric cancer patients; however, intrinsic or acquired resistance weakens the benefit of these approaches. Therefore, it is important to find ways to sensitize tumor cells to chemotherapy. Receptor tyrosine kinases (RTKs) can become abnormally activated and trigger downstream pathways to maintain tumor growth as well as tumor survival^17^,^18^. Moreover, a growing body of evidence has unveiled a correlation between EGFR-mediated mTORC2 activation and the resistance of gastric tumors to chemotherapy^19^,^20^. RTK blockade strategies have been shown to have a synergistic effect with chemotherapies in preclinical^21^ and clinical trials for gastric cancer^7^,^22^. Considering that angiogenesis plays a pivotal role in gastric cancer tumorigenesis^23^,^24^, we examined linifanib (ABT-869), a multi-RTK inhibitor^9^,^25^, to explore the benefit of ABT-869-containing strategies in the treatment of gastric cancer. Although ABT-869 is currently being examined in hepatocellular carcinoma clinical trials, no related preclinical or clinical studies have been carried out in gastric cancer. To the best of our knowledge, our study is the first to show the pronounced synergistic effect of ABT-869 and the first-line chemotherapeutic drugs (5-Fu and cisplatin) in human gastric cancers (CI ranging from 0.017 to 0.589). Moreover, the cisplatin-resistant cell line SGC-7901/DDP was also vulnerable to ABT-869-containing treatments, as drug co-treatments strengthened cell cycle arrest and enhanced cell death in these cisplatin-resistant gastric cancer cells (Fig. 2d). This effect may be attributed to diminished EGFR- and IGFR-mediated signaling pathways mediated by the ABT-869-containing therapy. Furthermore, angiogenesis inhibition by ABT-869 also contributed to the anti-neoplastic effects of the combined treatments. In a gastric cancer xenograft mouse model, ABT-869, in combination with either 5-Fu or cisplatin, blocked tumor growth and significantly prolonged mouse survival *in vivo* (Fig. 5). In addition, inhibition of the EGFR- and IGFR-mediated signaling pathways and angiogenesis were confirmed in the xenografts (Fig. 6). Our study is the first to show the validity that combining ABT-869 with chemotherapies results in a synergistic reduction in cell viability of resistant gastric cells *in vitro*, and an increased mouse survival *in vivo*.

Histologically, gastric cancer can be divided into two categories: diffuse gastric cancer (DGC) and intestinal gastric cancer (IGC). HER2 overexpression caused by ERBB2 amplification is more prevalent in IGC than in DGC^26^. Although abnormalities in HER2 occurs in approximately 15% of gastric cancer patients^27^, activation of its downstream pathways, such as RAS/MEK/MAPK and PI3K/AKT/mTOR, are reported to be more common in gastric cancer tumorigenesis^28^. Moreover, the PI3K/AKT/mTOR signaling pathway plays a pivotal role in primary and acquired resistance in gastric cancer^29^,^30^,^31^. Activation of the PI3K/AKT/mTOR cascade can be achieved by the interaction between HER2 and EGFR^32^ or IGFR^33^. Therefore, blockade of either EGFR or IGFR is thought to be beneficial for patients whose tumors response poorly to chemotherapy. Although we found that ABT-869 alone was insufficient to inhibit EGFR or IGFR activation that was consistent with previous findings^9^,^25^, the combination of ABT-869 and first-line chemotherapies exhibited a strong inhibitory effect on both EGFR and IGFR as well as the downstream AKT/mTOR cascade. The detailed mechanism underlying these effects awaits further investigation. In addition to its effects on cell survival, the selective anti-angiogenesis effect of ABT-869 confers an intriguing advantage in cancers that heavily depend on angiogenesis, including gastric cancer. Moreover, the important role of the AKT/mTOR pathway is further supported by its crosstalk with pathways involved in the regulation of stem cell fate^3^. This crosstalk is reported to be involved in the epithelial-mesenchymal transition (EMT) process^34^,^35^. Considering the emerging roles of the EMT in chemoresistance^36^,^37^, the effect of combined therapies containing ABT-869 on the EMT process should be further explored in gastric cancer.

Another important issue frequently raised in the clinic is the toxicity related to single targeted agent or combination therapy. Although 5-Fu- and cisplatin-based chemotherapies benefit the overall survival of gastric cancer patients, unbearable renal failure and other severe adverse effects make this standard treatment not appropriate for all patients^38^,^39^,^40^. Therefore, the use of combination therapy to enhance the efficacy of current chemotherapies is extremely important. In this study, ABT-869 potentiated the effects of 5-Fu and cisplatin in both *in vitro* and *in vivo* gastric cancer models, making it possible to reduce the dosage of current chemotherapies by using drug combination regimens containing ABT-869. Furthermore, our results showed that the combination of ABT-869 with either low-dose of 5-Fu or cisplatin did not lead to any obvious adverse effects *in vivo*, even after a 60-day treatment period. Unlike other multi-targeted RTK inhibitors, the selectivity of ABT-869 on VEGFR and PDGFR prevents many of adverse effects that typically result from RTK inhibition^9^,^25^. This is further supported by the finding that hepatocellular carcinoma patients are very tolerant to the ABT-869 treatment^12^,^41^.

In summary, we demonstrate that ABT-869 and chemotherapies act synergistically to inhibit the tumor growth and angiogenesis *in vitro* and *in vivo* through simultaneous blockade of EGFR-, IGF1R- and VEGR2-mediated AKT/mTOR signaling pathways. Given that ABT-869 is approved for use in clinical trials, our study provides a strong rationale for combining ABT-869 with current chemotherapeutic agents to treat gastric cancer patients.

## Methods

### Reagents and antibodies

ABT-869, 5-Fluorouracil (5-Fu) and cisplatin were purchased from Selleck Chemicals (Houston, TX). Recombinant human VEGF (VEGF_165_) was a gift from the Experimental Branch of the National Institutes of Health (Bethesda, MD). Recombinant human epidermal growth factor (EGF) and insulin-like growth factor (IGF) were purchased from R&D Systems (Minneapolis, MN). Growth factor-reduced Matrigel was obtained from BD Biosciences (San Diego, CA). The following antibodies were purchased from Cell Signaling Technology (Danvers, MA): VEGFR2, EGFR, IGF1R, AKT, mTOR, p70 S6K Kinase (S6K), eukaryotic initiation factor 4e (4EBP-1), poly (ADP-ribose) polymerase (PARP), Bcl-2, Bcl-xL, CyclinD1, phospho-specific anti-EGFR (Tyr^1068^), anti-IGF1R (Tyr^1316^), anti-VEGFR2 (Tyr^1175^), anti-AKT (Ser^473^), anti-mTOR (Ser^2448^), anti-S6K (Ser^235/236^) and anti-4EBP-1 (Thr^37/46^). Anti-CD31 antibody was obtained from Epitomics (Burlingame, CA).

### Cell lines and cell culture

Primary human umbilical vascular endothelial cells (HUVECs) were kindly provided by Dr. Xinli Wang (Department of Surgery, Baylor College of Medicine, Houston, TX). HUVECs were cultured in endothelial cell growth medium as described previously^42^,^43^,^44^: M199 medium (Gibco, Invitrogen) supplemented with 20% fetal bovine serum (FBS, Hyclone Laboratories, Logan, UT), 20 μg/mL bovine endothelial cell growth factor (Roche Life Science), 0.1 mg/mL Heparin (Sigma), 15 mmol/L HEPES (4-(2-hydroxyethyl)-1-piperazineethanesulfonic acid) buffer, penicillin (50 IU/L), streptomycin (50 mg/L), NaHCO_3_ (44 mmol/L) and 50 μg/mL amphotericin B. Prior written informed consent was obtained from all patients, and the study protocol was approved by the ethics committee of Baylor College of Medicine. The methods for HUVEC isolation were carried out in accordance with the NIH standards established for the human cell lines, and all of the experimental protocols on HUVECs were approved by the committee of Baylor College of Medicine and East China Normal University. The AGS gastric cancer cell line was purchased from the American Type Culture Collection (Manassas, VA). The SGC-7901, MGC-803 and BGC-823 cancer cell lines were obtained from the China Center for Type Culture Collection (Shanghai, China). The multidrug-resistant gastric cancer cell line SGC-7901/DDP was purchased from Sciencell (Shanghai, China) and was routinely maintained in media containing 10 μg/mL diamminedichloroplatinum (Sigma, St. Louis, MO). All these gastric cancer cell lines were cultured in RPMI-1640 medium supplemented with 10% FBS (HyClone Laboratories). A normal human gastric epithelial cell line (GES-1 cells) was also purchased from the China Center for Type Culture Collection and cultured in Dulbecco’s Modified Eagle’s Medium, supplemented with 10% FBS (HyClone Laboratories). The above cells were authenticated before use by short tandem repeat analysis, and cultured at 37 °C under a humidified 95%:5% (v/v) mixture of air and CO_2_.

### Cell viability assay

A cell viability assay was performed using the CellTiter 96^®^ AQueous One Solution Cell Proliferation kit (Promega, Madison, WI). The half inhibitory concentration (IC_50_) values were calculated using Prism 5 (GraphPad Software, La Jolla, CA). The combination index (CI) equation, described by Chou-Talalay^45^, was generated using CalcuSyn software (Version 2; Biosoft). Combination treatments with CIs < 1 were considered to be synergistic. We determined the Fa (fraction affected by the dose) and CI values of each drug pair. All of the experiments were independently repeated three times.

### Cell cycle analysis

After the cancer cells were treated with various drug combinations, they were fixed and re-suspended in a propidium iodide (PI)/RNase staining buffer (Sigma). The stained cells were then analyzed for DNA content using a FACScan cell analyzer equipped with Cellquest software (BD Biosciences).

### Apoptosis analysis

To detect synergistic effects of the chemotherapeutic drugs on cell apoptosis, treated cells were analyzed using the FITC-Annexin V apoptosis kit (BD Biosciences, San Jose, CA). The results were analyzed by flow cytometry (FACSCalibur, BD Science). All of the experiments were independently repeated three times.

### Live/dead staining assay

Apoptosis was examined in SGC-7901/DDP cells using a live/dead reagent (Invitrogen, Carlsbad, CA), which measured the intracellular esterase activity and plasma membrane integrity.

### Transwell migration assay

Endothelial cell migration was assayed in Boyden chambers (BD Biosciences), as previously described^46^. Photographs of four random fields were taken using an Olympus inverted microscope, and the cells were quantified by manual counting based on untreated control wells. Three independent experiments were performed.

### Animal studies

C57BL/6 mice and BALB/cA nude mice used in the present study were purchased from National Rodent Laboratory Animal Resources (Shanghai, China) and maintained in a laminar airflow cabinet under specific pathogen-free conditions and a 12 h light–dark cycle. All treatments were administered according to the NIH standards established in the Guidelines for the Care and Use of Experimental Animals. All of the experimental protocols were approved by the Animal Investigation Committee of East China Normal University.

### Matrigel plug assay

Six to 8-week-old C57BL/6 mice were used in the Matrigel plug assay. As described previously^47^, Matrigel in the presence or absence of 50 ng VEGF and 20 units of heparin, plus the addition of ABT-869 (0.1 μmol/L) and/or 5-Fu (10 μmol/L) and/or cisplatin (15 μmol/L) was subcutaneously injected into the ventral area of C57/BL/6 mice. Seven days after the implantation, the intact matrigel plugs were carefully removed and imaged.

### Human gastric tumor xenograft mouse model

Four to 6-week-old nude mice were used to set up gastric tumor xenograft mouse model. Briefly, SGC-7901 cancer cells (3.5 × 10^6^ cells/mouse) were subcutaneously injected into the right flank of each mouse. After 9 days, when the average tumor size reached approximately 100 mm^3^, the mice were randomized into six groups (n = 10–11) and orally gavaged with ABT-869 (10 mg/kg/day) and/or intraperitoneal injected (i.p.) with 5-Fu (10 mg/kg/day) or cisplatin (2 mg/kg/day) at five-day intervals for eight cycles. The dosage of each drug was determined according to published literatures. An equivalent amount of pure refined corn oil served as a vehicle control. The tumor volumes of the xenografts were measured with calipers using the following formula: V = (L × [W]^2^) × 0.52; where L is the longest diameter of the tumor and W is the shortest diameter of the tumor. Twenty-six days after the initiation of treatment, tumor-bearing mice were imaged and a parallel western blotting experiment was performed to determine the phosphorylation levels of EGFR, IGFR and AKT, as well as their total protein levels (using 2 randomly selected tumor samples from each group). For long-term survival studies, animals were sacrificed when the tumor volumes reached 1,800 mm^3^. Tumor xenograft tissue was further processed for immunohistochemistry analysis using p-EGFR (Tyr^1068^), p-AKT (Ser^473^), Ki-67 and CD31 antibodies. Images were taken using a Leica DM 4000B photo microscope (Solms, Germany; magnification, 400x).

### Western blotting analysis

The cancer cells were treated with the specified drugs and lysed in cold radioimmunoprecipitation assay buffer containing a proteinase inhibitor cocktail. The protein concentrations were determined using the Pierce BCA Protein Assay Kit (Pierce Biotechnology, Rockford, IL) and equalized prior to loading. Approximately 40–50 μg of cellular protein from cells in each treatment group was applied to 6% or 10% SDS-polyacrylamide gels and subsequently transferred onto nitrocellulose membranes (Millipore, Billerica, MA). The signals were detected using the Li-Cor Odyssey Infrared system (LI-COR Biosciences, Lincoln, NE).

### Statistical analysis

The data are presented as the mean ± standard deviation. Statistical tests were performed using Microsoft Excel and GraphPad Prism Software version 5.0 (GraphPad Software Inc., San Diego, CA). For two-group comparisons, a two-tailed unpaired *t*-test was used. For multiple group comparisons, one-way ANOVA analysis was used. A value of *P* < 0.05 was considered to be significant. Survival curves were analyzed using the log-rank test (Kaplan-Meier survival curves).

## Additional Information

**How to cite this article**: Chen, J. *et al*. Linifanib (ABT-869) Potentiates the Efficacy of Chemotherapeutic Agents through the Suppression of Receptor Tyrosine Kinase-Mediated AKT/mTOR Signaling Pathways in Gastric Cancer. *Sci. Rep.* **6**, 29382; doi: 10.1038/srep29382 (2016).

## Supplementary Material

Supplementary Information

## References

[b1] Wang K. . Whole-genome sequencing and comprehensive molecular profiling identify new driver mutations in gastric cancer. Nat Genet 46, 573–582 (2014). 24816253 10.1038/ng.2983

[b2] Shah M. A. . Molecular classification of gastric cancer: a new paradigm. Clin Cancer Res 17, 2693–2701 (2011). 21430069 10.1158/1078-0432.CCR-10-2203 PMC3100216

[b3] Wadhwa R. . Gastric cancer-molecular and clinical dimensions. Nat Rev Clin Oncol 10, 643–655 (2013). 24061039 10.1038/nrclinonc.2013.170 PMC3927982

[b4] Potente M., Gerhardt H. & Carmeliet P. Basic and therapeutic aspects of angiogenesis. Cell 146, 873–887 (2011). 21925313 10.1016/j.cell.2011.08.039

[b5] Chen J. . Prognostic significance of vascular endothelial growth factor expression in gastric carcinoma: a meta-analysis. J Cancer Res Clin Oncol 137, 1799–1812 (2011). 21918901 10.1007/s00432-011-1057-2 PMC11827832

[b6] Van Cutsem E. . Bevacizumab in combination with chemotherapy as first-line therapy in advanced gastric cancer: a biomarker evaluation from the AVAGAST randomized phase III trial. J Clin Oncol 30, 2119–2127 (2012). 22565005 10.1200/JCO.2011.39.9824

[b7] Sun W. . Phase II study of sorafenib in combination with docetaxel and cisplatin in the treatment of metastatic or advanced gastric and gastroesophageal junction adenocarcinoma: ECOG 5203. J Clin Oncol 28, 2947–2951 (2010). 20458043 10.1200/JCO.2009.27.7988 PMC2903332

[b8] Dai Y. . Discovery of N-(4-(3-amino-1H-indazol-4-yl)phenyl)-N′-(2-fluoro-5-methylphenyl)urea (ABT-869), a 3-aminoindazole-based orally active multitargeted receptor tyrosine kinase inhibitor. J Med Chem 50, 1584–1597 (2007). 17343372 10.1021/jm061280h

[b9] Albert D. H. . Preclinical activity of ABT-869, a multitargeted receptor tyrosine kinase inhibitor. Mol Cancer Ther 5, 995–1006 (2006). 16648571 10.1158/1535-7163.MCT-05-0410

[b10] Shankar D. B. . ABT-869, a multitargeted receptor tyrosine kinase inhibitor: inhibition of FLT3 phosphorylation and signaling in acute myeloid leukemia. Blood 109, 3400–3408 (2007). 17209055 10.1182/blood-2006-06-029579 PMC1852258

[b11] Jasinghe V. J. . ABT-869, a multi-targeted tyrosine kinase inhibitor, in combination with rapamycin is effective for subcutaneous hepatocellular carcinoma xenograft. J Hepatol 49, 985–997 (2008). 18930332 10.1016/j.jhep.2008.08.010

[b12] Zhou J. . *In vivo* activity of ABT-869, a multi-target kinase inhibitor, against acute myeloid leukemia with wild-type FLT3 receptor. Leuk Res 32, 1091–1100 (2008). 18160102 10.1016/j.leukres.2007.11.025

[b13] Linifanib. Drugs R D 10, 111–122 (2010). 20698720 10.2165/11584520-000000000-00000 PMC3586091

[b14] Zhou J. . Synergistic antileukemic effects between ABT-869 and chemotherapy involve downregulation of cell cycle-regulated genes and c-Mos-mediated MAPK pathway. Leukemia 22, 138–146 (2008). 17943175 10.1038/sj.leu.2404960

[b15] Dun J. . Resveratrol synergistically augments anti-tumor effect of 5-FU *in vitro* and *in vivo* by increasing S-phase arrest and tumor apoptosis. Exp Biol Med (Maywood) (2015). 10.1177/1535370215573396 PMC4935345 25736303

[b16] Nishio K. & Saijo N. [Effect of cisplatin on cell cycle regulators]. Gan To Kagaku Ryoho 21, 289–294 (1994). 8109984

[b17] Yasumoto K. . The EGFR ligands amphiregulin and heparin-binding egf-like growth factor promote peritoneal carcinomatosis in CXCR4-expressing gastric cancer. Clin Cancer Res 17, 3619–3630 (2011). 21482691 10.1158/1078-0432.CCR-10-2475

[b18] Terashima M. . Impact of expression of human epidermal growth factor receptors EGFR and ERBB2 on survival in stage II/III gastric cancer. Clin Cancer Res 18, 5992–6000 (2012). 22977193 10.1158/1078-0432.CCR-12-1318

[b19] Navolanic P. M., Steelman L. S. & McCubrey J. A. EGFR family signaling and its association with breast cancer development and resistance to chemotherapy (Review). Int J Oncol 22, 237–252 (2003). 12527919

[b20] Tanaka K. . Oncogenic EGFR signaling activates an mTORC2-NF-kappaB pathway that promotes chemotherapy resistance. Cancer Discov 1, 524–538 (2011). 22145100 10.1158/2159-8290.CD-11-0124 PMC3229221

[b21] Laurila N. & Koivunen J. P. EGFR inhibitor and chemotherapy combinations for acquired TKI resistance in EGFR-mutant NSCLC models. Med Oncol 32, 205 (2015). 26081015 10.1007/s12032-015-0627-6

[b22] Macdonald D. A. . A phase I/II study of sorafenib in combination with low dose cytarabine in elderly patients with acute myeloid leukemia or high-risk myelodysplastic syndrome from the National Cancer Institute of Canada Clinical Trials Group: trial IND.186. Leuk Lymphoma 54, 760–766 (2013). 23061485 10.3109/10428194.2012.737917

[b23] Metges J. P. . Angiogenesis and p53 status in gastric cancer: A prospective serum and immunohistochemical study. Annals of Oncology 11, 64–64 (2000).

[b24] Barzi A. & Thara E. Angiogenesis in esophageal and gastric cancer: a paradigm shift in treatment. Expert Opinion on Biological Therapy 14, 1319–1332 (2014). 24881691 10.1517/14712598.2014.921677

[b25] Aversa C. . Linifanib: current status and future potential in cancer therapy. Expert Rev Anticancer Ther 15, 677–687 (2015). 25936222 10.1586/14737140.2015.1042369

[b26] Zhang X. L. . Comparative study on overexpression of HER2/neu and HER3 in gastric cancer. World J Surg 33, 2112–2118 (2009). 19636613 10.1007/s00268-009-0142-z

[b27] Ieni A. . HER2 status in advanced gastric carcinoma: A retrospective multicentric analysis from Sicily. Oncol Lett 6, 1591–1594 (2013). 24260051 10.3892/ol.2013.1611 PMC3833944

[b28] Isinger-Ekstrand A. . Genetic profiles of gastroesophageal cancer: combined analysis using expression array and tiling array–comparative genomic hybridization. Cancer Genet Cytogenet 200, 120–126 (2010). 20620594 10.1016/j.cancergencyto.2010.03.013

[b29] Brognard J., Clark A. S., Ni Y. & Dennis P. A. Akt/protein kinase B is constitutively active in non-small cell lung cancer cells and promotes cellular survival and resistance to chemotherapy and radiation. Cancer Res 61, 3986–3997 (2001). 11358816

[b30] Tsurutani J., West K. A., Sayyah J., Gills J. J. & Dennis P. A. Inhibition of the phosphatidylinositol 3-kinase/Akt/mammalian target of rapamycin pathway but not the MEK/ERK pathway attenuates laminin-mediated small cell lung cancer cellular survival and resistance to imatinib mesylate or chemotherapy. Cancer Res 65, 8423–8432 (2005). 16166321 10.1158/0008-5472.CAN-05-0058

[b31] Chen C. T. . MET activation mediates resistance to lapatinib inhibition of HER2-amplified gastric cancer cells. Mol Cancer Ther 11, 660–669 (2012). 22238368 10.1158/1535-7163.MCT-11-0754 PMC4209288

[b32] Terashima M. . Impact of Expression of Human Epidermal Growth Factor Receptors EGFR and ERBB2 on Survival in Stage II/III Gastric Cancer. Clinical Cancer Research 18, 5992–6000 (2012). 22977193 10.1158/1078-0432.CCR-12-1318

[b33] Browne B. C. . Inhibition of IGF1R activity enhances response to trastuzumab in HER-2-positive breast cancer cells. Ann Oncol 22, 68–73 (2011). 20647220 10.1093/annonc/mdq349

[b34] Chen X. . mTOR regulate EMT through RhoA and Rac1 pathway in prostate cancer. Mol Carcinog 54, 1086–1095 (2015). 25043657 10.1002/mc.22177

[b35] Kwasnicki A., Jeevan D., Braun A., Murali R. & Jhanwar-Uniyal M. Involvement of mTOR signaling pathways in regulating growth and dissemination of metastatic brain tumors via EMT. Anticancer Res 35, 689–696 (2015). 25667447

[b36] Sarkar F. H., Li Y., Wang Z. & Kong D. Pancreatic cancer stem cells and EMT in drug resistance and metastasis. Minerva Chir 64, 489–500 (2009). 19859039 PMC2878773

[b37] Singh A. & Settleman J. EMT, cancer stem cells and drug resistance: an emerging axis of evil in the war on cancer. Oncogene 29, 4741–4751 (2010). 20531305 10.1038/onc.2010.215 PMC3176718

[b38] Offerman J. J. . Acute effects of cis-diamminedichloroplatinum (CDDP) on renal function. Cancer Chemother Pharmacol 12, 36–38 (1984). 6537801 10.1007/BF00255906

[b39] Kurokawa Y. . [Efficacy and side effect of continuous intra-arterial infusion of high-dose 5-FU for liver metastases of colorectal cancer]. Gan To Kagaku Ryoho 26, 1737–1740 (1999). 10560384

[b40] Suzuki H. . [Renal-salt wasting syndrome in a patient with CDDP containing chemotherapy for recurrent non-small-cell lung cancer]. Gan To Kagaku Ryoho 38, 2635–2638 (2011). 22189232

[b41] Toh H. C. . Phase 2 trial of linifanib (ABT-869) in patients with unresectable or metastatic hepatocellular carcinoma. Cancer 119, 380–387 (2013). 22833179 10.1002/cncr.27758

[b42] Pang X. . Acetyl-11-keto-beta-boswellic acid inhibits prostate tumor growth by suppressing vascular endothelial growth factor receptor 2-mediated angiogenesis. Cancer Res 69, 5893–5900 (2009). 19567671 10.1158/0008-5472.CAN-09-0755 PMC2724674

[b43] Wang J., Wilcken D. E. & Wang X. L. Cigarette smoke activates caspase-3 to induce apoptosis of human umbilical venous endothelial cells. Molecular genetics and metabolism 72, 82–88 (2001). 11161833 10.1006/mgme.2000.3115

[b44] Senthil D. . Genotype-dependent expression of endothelial nitric oxide synthase (eNOS) and its regulatory proteins in cultured endothelial cells. DNA and cell biology 24, 218–224 (2005). 15812238 10.1089/dna.2005.24.218 PMC1350115

[b45] Chou T. C. Theoretical basis, experimental design, and computerized simulation of synergism and antagonism in drug combination studies. Pharmacological reviews 58, 621–681 (2006). 16968952 10.1124/pr.58.3.10

[b46] Wang J. . Small molecule 1′-acetoxychavicol acetate suppresses breast tumor metastasis by regulating the SHP-1/STAT3/MMPs signaling pathway. Breast Cancer Res Treat 148, 279–289 (2014). 25301089 10.1007/s10549-014-3165-6

[b47] Pang X. . Morelloflavone, a biflavonoid, inhibits tumor angiogenesis by targeting rho GTPases and extracellular signal-regulated kinase signaling pathways. Cancer Res 69, 518–525 (2009). 19147565 10.1158/0008-5472.CAN-08-2531 PMC2662342

