# Supplementary material for: Linifanib (ABT-869) Potentiates the Efficacy of Chemotherapeutic Agents through the Suppression of Receptor Tyrosine Kinase-Mediated AKT/mTOR Signaling Pathways in Gastric Cancer
Source: Sci Rep. 2016 Jul 8;6:29382. doi: 10.1038/srep29382 (PMC4937412; doi:10.1038/srep29382)
Supplement: Supplementary Information [file srep29382-s1.pdf]

Supplementary information for

Linifanib (ABT-869) Potentiates the Efficacy of Chemotherapeutic Agents through the Suppression of Receptor Tyrosine Kinase-Mediated AKT/mTOR Signaling Pathways in Gastric Cancer

Jing Chen[1,2,$], Jiawei Guo[1,$], Zhi Chen[1], Jieqiong Wang[1,3], Mingyao Liu[1,4], Xiufeng Pang[1,*]

[1] Shanghai Key Laboratory of Regulatory Biology, Institute of Biomedical Sciences and School of Life Sciences, East China Normal University, Shanghai 200241, China; [2] Key Laboratory of Reproduction and Genetics in Ningxia, Ningxia Medical University, Yinchuan 750004, China; [3] Cancer Institute, Fudan University Shanghai Cancer Center; Department of Oncology, Shanghai Medical College, Fudan University, Shanghai 200032, China; [4] Institute of Biosciences and Technology, Department of Molecular and Cellular Medicine, Texas A&M University Health Science Center, Houston, Texas 77030, USA.

*Correspondence:

Xiufeng Pang, Ph.D

Phone: +86-21-24206942; Fax: +86-21-54344922; E-mail: xfpang@bio.ecnu.edu.cn

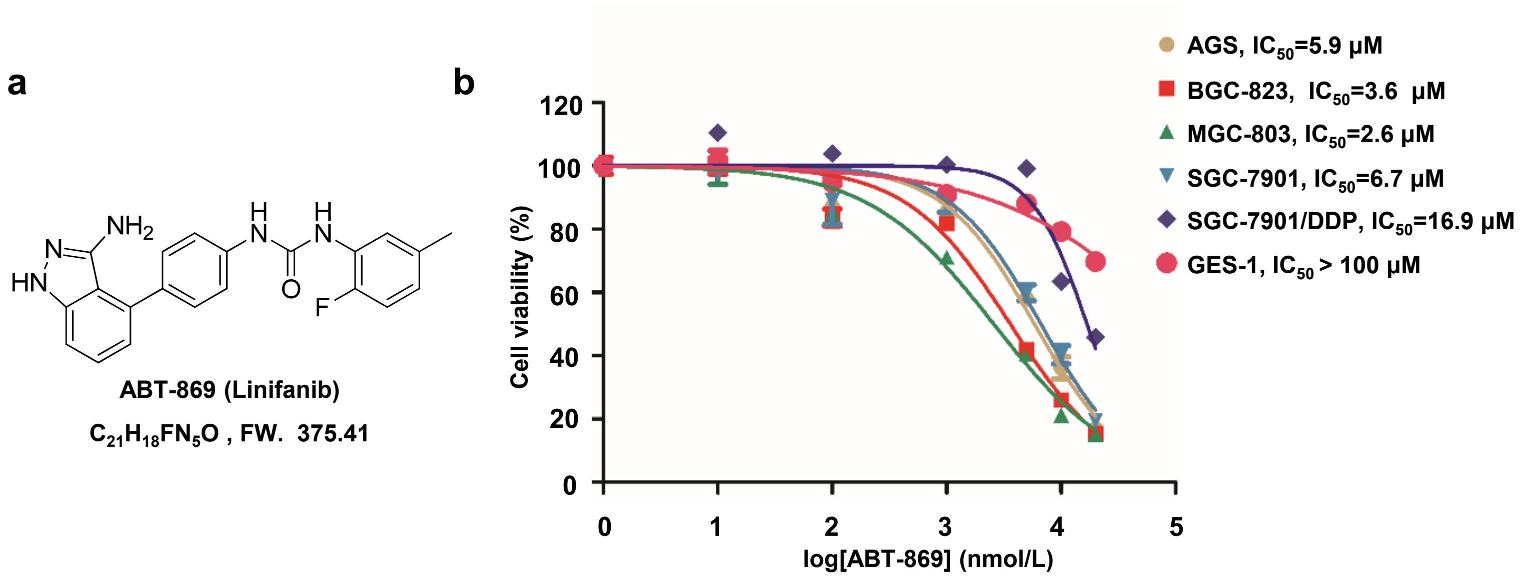

Figure S1. The cytotoxicity of ABT-869 in gastric cancer cells. ***a.*** The chemical structure of ABT-869. ***b.*** The cytotoxicity of ABT-869 in a broad of gastric cancer cell lines. After the cells were treated with ABT-869 for 48 h, cell viability assays were performed. The half maximal inhibitory concentration (IC_{50}) was calculated by Prism 5 (GraphPad Software, La Jolla, CA). *Dots*, mean of five replicates.

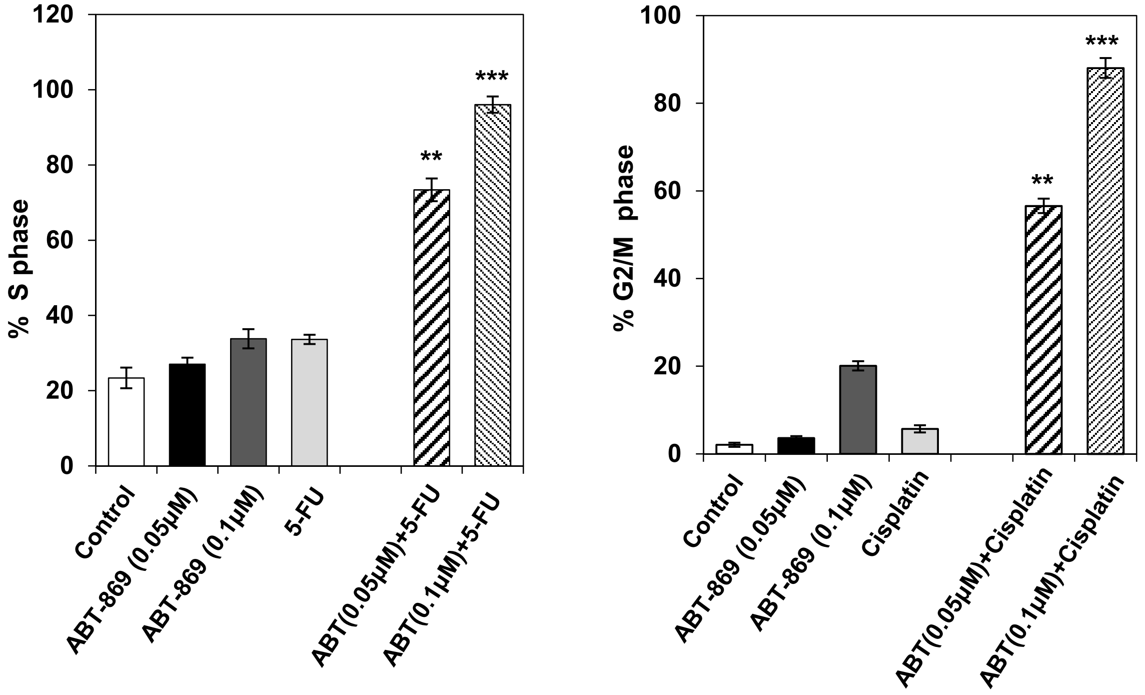

Figure S2. ABT-869 potentiates cell cycle arrest induced by 5-Fu or cisplatin in MGC-803 gastric cancer cells. MGC-803 cells were treated with indicated concentrations of agents (15 μmol/L of 5-FU; 10 μmol/L of cisplatin; 0.05 μmol/L or 0.1 μmol/L of ABT-869) either alone or in combination for 48 h, followed by cell cycle analysis. *Columns*, mean; *bars*, standard deviation. **, $P < 0.01$; ***, $P < 0.001$ *vs.* 5-FU or cisplatin alone group.

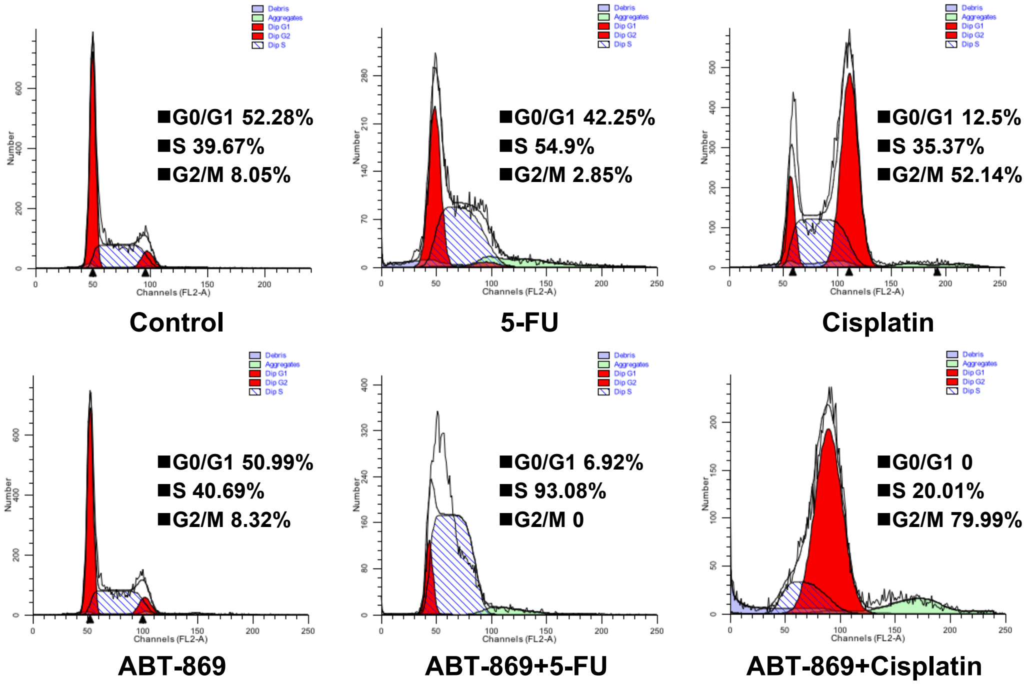

Figure S3. ABT-869 potentiates cell cycle arrest induced by 5-Fu or cisplatin in BGC-823 gastric cancer cells. BGC-823 cells were treated with indicated drugs (15 μmol/L of 5-FU, 10 μmol/L of Cisplatin and 0.1 μmol/L of ABT-869) either alone or in combination for 48 h, followed by cell cycle analysis. BGC-823 gastric cancer cells were obtained from the China Center for Type Culture Collection (Shanghai, China).

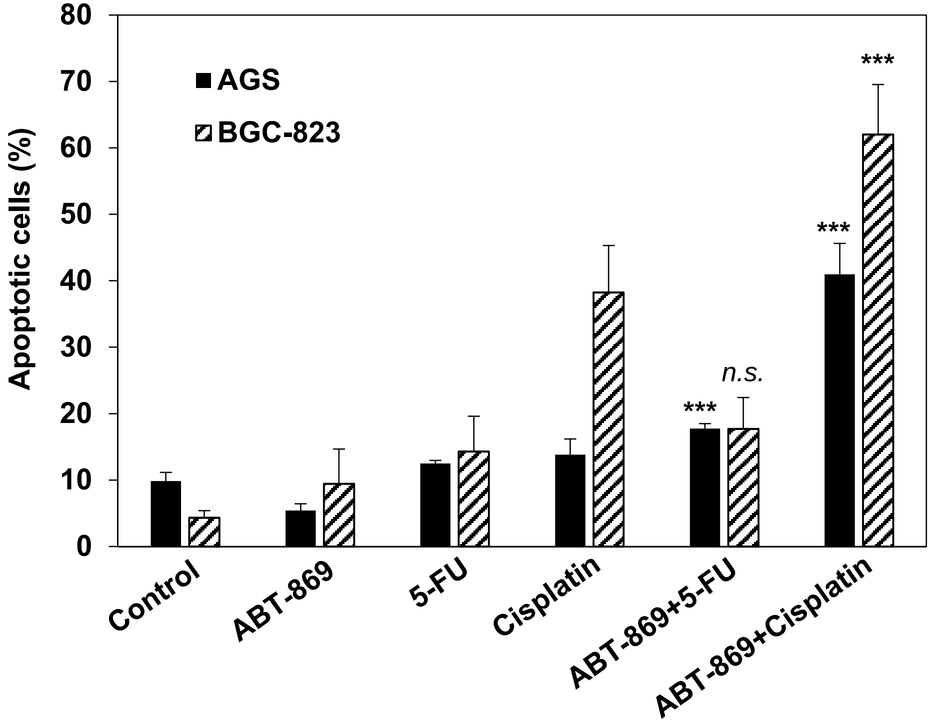

Figure S4. ABT-869 potentiates apoptosis induced by 5-Fu or cisplatin in AGS and BGC-823 gastric cancer cells. AGS and BGC-823 cells were treated with indicated drugs (15 μmol/L of 5-FU, 10 μmol/L of cisplatin, 0.1 μmol/L of ABT-869) either alone or in combination for 48 h, followed by cell apoptosis determination. AGS gastric cancer cells were obtained from the China Center for Type Culture Collection (Shanghai, China). *Columns*, mean; *bars*, standard deviation. ***, $P < 0.001$ *vs.* 5-FU or cisplatin alone group.
